# Comparative finite element modelling of surgical meshes at macro and mesoscale: assessing the role of morphology on mechanical behavior

**DOI:** 10.1007/s11517-026-03645-6

**Published:** 2026-08-06

**Authors:** Vittoria Civilini, Alessandra Aldieri, Dario Carbonaro, Vincenzo Giacalone, Alberto L. Audenino, Mara Terzini

**Affiliations:** 1 Department of Mechanical and Aerospace Engineering, Politecnico di Torino, Corso Duca degli Abruzzi, 24, Turin, 10129 Italy; 2 Polito BIO Med Lab, Politecnico di Torino, Turin, 10129 Italy; 3 Department of Industrial Engineering, Alma Mater Studiorum - University of Bologna, Bologna, 40126 Italy; 4 Department of Mechanical, Energy and Management Engineering, Università della Calabria, Rende, 87030 Italy

**Keywords:** Mesoscale FE models, Surgical mesh, Textile, Pore deformation, Hyperelastic textile

## Abstract

Synthetic meshes are widely used in abdominal wall surgery, yet recurrences remain common. Understanding how mesh morphology evolves under physiological loads is therefore crucial. In this work, a mesoscale finite element approach was developed and compared against the conventional macroscale approach to investigate the mechanical response of lightweight and standard weight polypropylene meshes using previously performed experimental tests. The new mesoscale approach reconstructed the porous architecture from high-resolution images, allowing anisotropy to emerge from geometry while using an isotropic hyperelastic material formulation. Macroscale models were implemented for comparison, modelling meshes as homogeneous surfaces with an anisotropic hyperelastic formulation. Material properties in both approaches were calibrated on uniaxial tensile tests and evaluated through ball burst simulations. Both strategies showed comparable agreement with experiments at the surgical mesh-level. However, mesoscale models provided insights into pore-level mechanisms, including deformation and collapse, confirmed through image-based tracking. They also confirmed that the observed anisotropy can be reproduced numerically by reconstructing the textile architecture. In conclusion, while the macroscale approach is faster to implement, mesoscale models better capture how structural features influence pore-level mechanical response. Integrating this approach into more complex simulations could help predict in vivo performance and guide the design of next-generation surgical meshes.

**Graphical abstract:**

**Figure:**
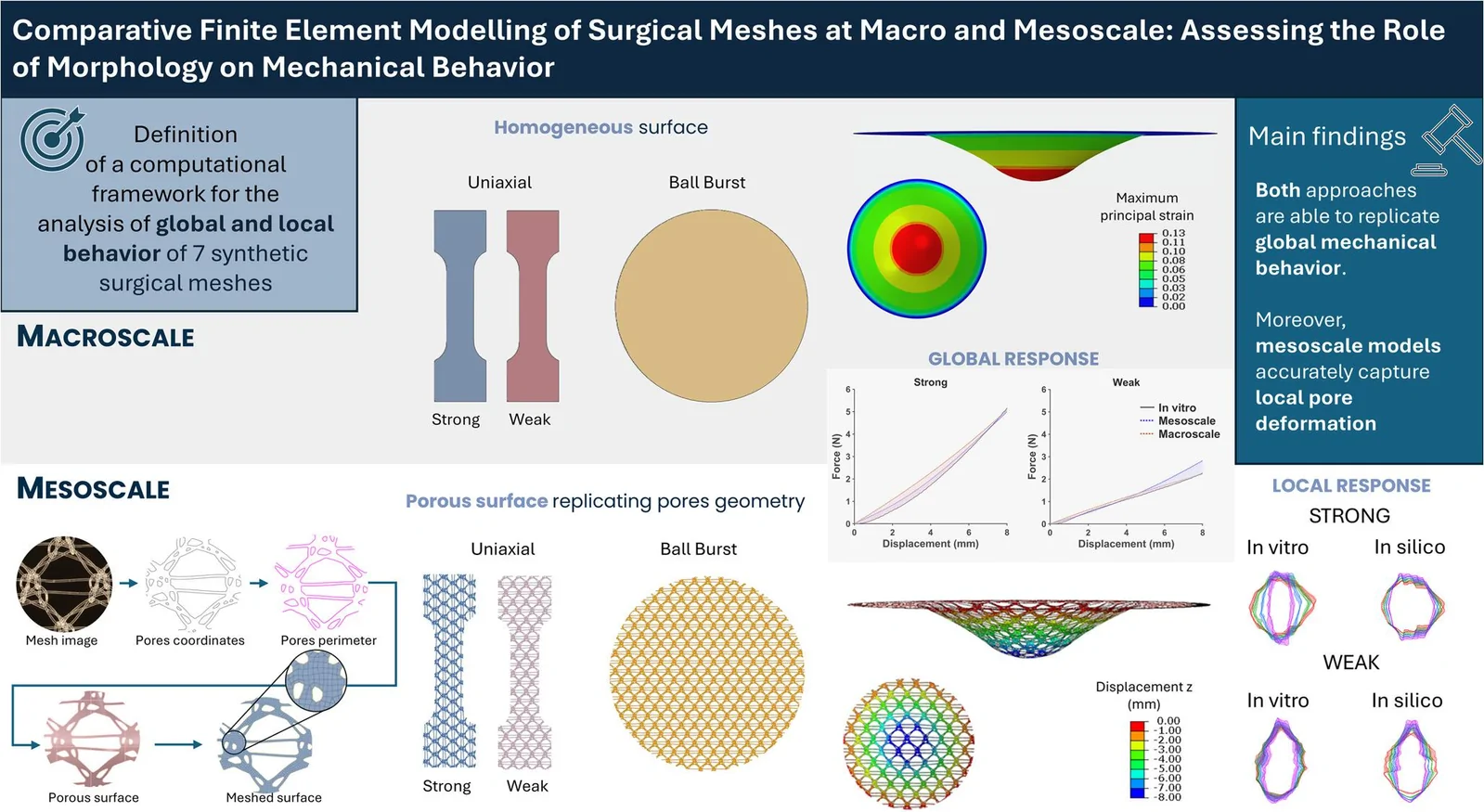

**Supplementary Information:**

The online version contains supplementary material available at https://doi.org/10.1007/s11517-026-03645-6.

## Introduction

Synthetic meshes are widely used in the treatment of abdominal and inguinal hernia. Over the last few decades, various knitted patterns have been developed, trying to improve surgical outcomes [1]. However, recurrence rates remain high, reaching almost 30% during the five years post-surgery [2]. The knitted pattern and aspects such as pore shape and dimensions, more specifically, play a crucial role in tissue integration and, therefore, affect the rate of recurrence [3–5]. Nevertheless, after implantation, physiological loading conditions can lead to pore collapse, i.e. a marked reduction in pore opening and effective pore area due to structural deformation of the mesh, resulting in a decrease in effective porosity and potentially compromising tissue integration and overall performance [6–10]. At present, in vitro tests used to characterize hernia meshes typically provide information based on highly simplified loading scenarios (e.g., uniaxial or biaxial tension), which significantly differ from the in vivo solicitations these devices face after implantation [11, 12]. In this perspective, validated finite element (FE) models of these meshes provide useful insights, enabling the analysis of multidirectional and multifaceted loads as well as the integration with abdominal FE models. Textiles have commonly been simulated at different length scales: macroscale, mesoscale, and microscale, each offering distinct advantages in terms of accuracy, computational cost, and their ability to capture local deformation mechanisms. In particular, macroscale models, despite neglecting the textile architecture, often provide a good representation of the surgical mesh-level response after calibration and could be used to simulate larger patches [13, 14]. By contrast, microscale models provide the highest level of fidelity by resolving individual fibers and their interactions, but at the cost of significantly higher computational complexity and limited applicability to large structures [15, 16]. Mesoscale models offer a good balance between detail and efficiency, as they explicitly represent the yarn or pore architecture and allow for capturing local deformation mechanisms [17–19]. Although FE models have been widely used in the literature for textile devices across different fields, they are mostly implemented at the macroscale in the biomedical context, where devices are usually warp-knitted [20–22]. To replicate the anisotropic nonlinear mechanical behavior of these devices, these macroscale models have commonly been implemented using various constitutive models, e.g., anisotropic hyperelastic constitutive models usually used for soft tissues [23, 24], anisotropic hypo-elastoplastic models [13], bilinear orthotropic models [25]. As mentioned before, these FE models allow the study of the overall mechanical behavior of the mesh with low computational cost, but they completely overlook the local phenomena, such as pore deformation, collapse, and morphological changes that are typical of warp-knitted structures. Nonetheless, as pores shape and dimension strongly affect the mechanical behaviour, the analysis of pores deformation under complex loads could give important information about the tissue integration and the in vivo performance [26, 27]. In this regard, mesoscale models provide a more sophisticated replica of the meshes, despite being often limited by the need to find a compromise between simplicity and faithful reconstruction. Most of the existing mesoscale models focus on macroporous lightweight meshes or urogynecologic devices, which typically exhibit a simple knitted architecture [6, 28–32]. Mesoscale models reported in the literature adopted different simplification approaches: bundles of yarns may be simplified into single beam elements [29, 32], or a more detailed description of small portions of mesh patches may be done through solid elements [33]. Regarding the interaction between yarns, in these approaches yarn relative displacement is constrained. An alternative approach based on the theory of multibody systems was proposed by Röhrnbauer et al., 2014. It was applied on a unit cell of a urogynecologic mesh, using line and force elements that allow the study of nonlinearity, although limiting the simulation to only half a pore and only under simple and uniform loads. The described approaches have limited applicability on standard weight meshes, which, although less frequently implanted compared to lightweight ones, are still successfully used in clinical practice [34]. Mesoscale models developed for auxetic textiles, in contrast, demonstrate the potential to incorporate porous architecture even in denser structures, offering a more detailed representation of mechanical behaviour [17, 35, 36]. In this scenario, a mesoscale modeling approach capable of representing both lightweight and standard weight meshes is currently missing.

Therefore, in this study, we propose a mesoscale modeling approach able to capture pore-level mechanics across mesh types, including lightweight (LW) and standard weight (SW) variants. The approach performance was compared to conventional macroscale models of the same meshes to highlight the strengths and limitations of each approach systematically.

## Materials and methods

**Fig. 1:**
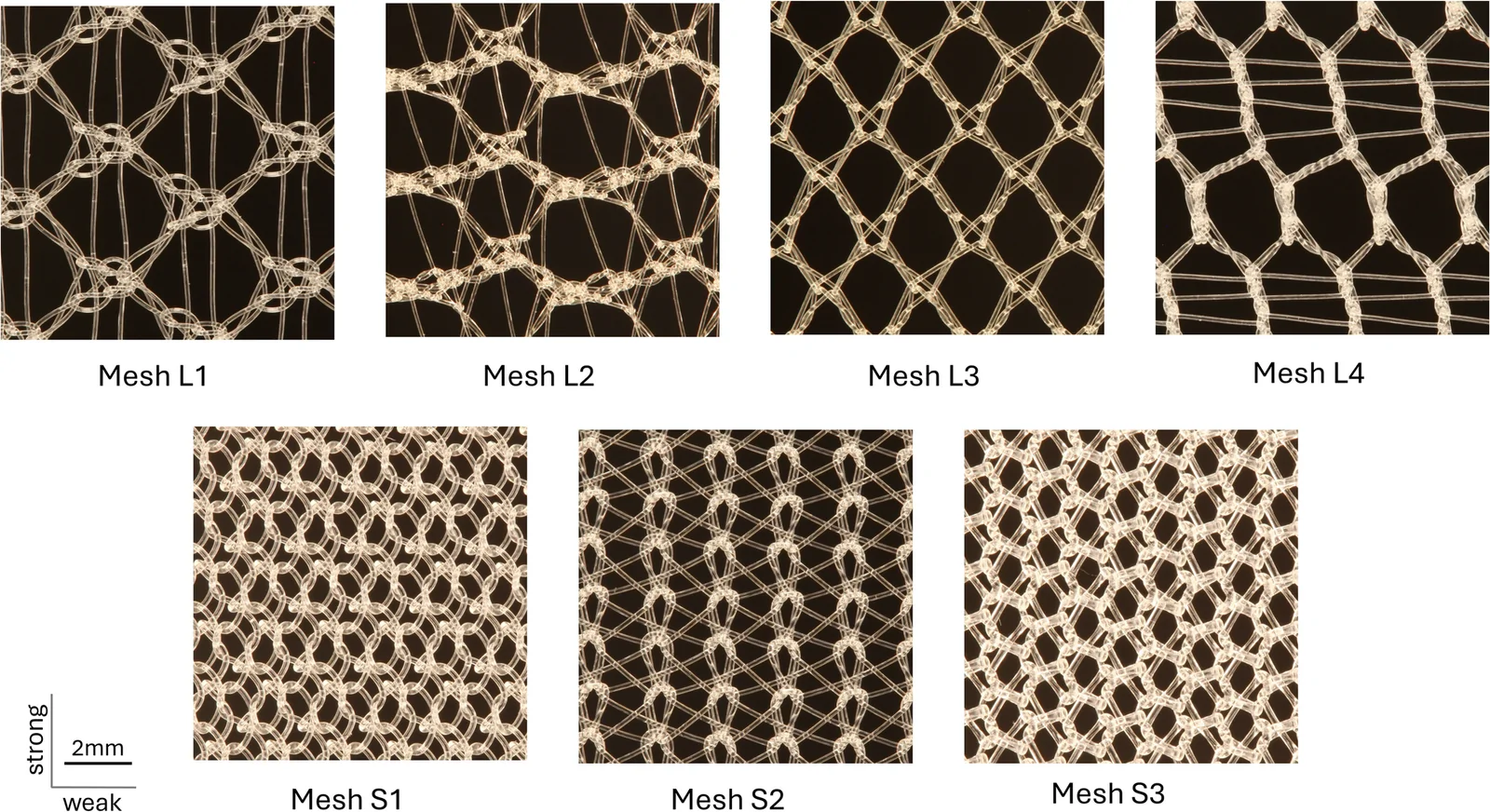
The seven synthetic surgical meshes addressed in this study, oriented according to the strong and the weak directions. The first row contains the LW meshes (L1–4), while the second row contains the SW meshes (S1–3)

In this study, seven commercial synthetic polypropylene surgical meshes were considered (Fig. 1). The seven synthetic surgical meshes addressed in this study were oriented according to the strong and the weak directions. Specifically, four meshes were classified as lightweight (L1–4), while three were standard-weight (S1–3). Table 1 reports the main morphological parameters typically used to characterize surgical meshes in the literature, including thickness, areal density (weight), and textile porosity. All these meshes had been previously mechanically characterized in our laboratory, as presented in Civilini et al., 2023, through uniaxial and ball-burst experimental tests [37].

**Table 1:** Morphological characteristics of the seven meshes modelled in the study. The column corresponding to the thickness represents the nominal thickness of the meshes used in the macroscale models and in the yarn bundle region of the mesoscale models

Mesh ID	Thickness (mm)	Weight (g/m^2^)	Textile porosity (%)
L1	0.42	40.34	67.20
L2	0.45	45.74	63.50
L3	0.37	35.53	69.37
L4	0.62	50.38	66.09
S1	0.71	110.55	42.08
S2	0.52	76.79	49.83
S3	0.56	132.41	37.62

The development of the FE models for the seven meshes consisted of three main phases:

1. The creation of each mesh of two FE models replicating (i) a dogbone specimen and (ii) a circular specimen, which were adopted for the uniaxial tensile test and for the ball burst test, respectively.
2. The calibration of material model parameters with experimental force-displacement data derived from the uniaxial tests.
3. The validation of the calibrated FE models with experimental force-displacement data derived from ball burst tests.

The subsequent sections describe the development of the mesoscale and macroscale FE models, with a focus on the differences between the two approaches in terms of geometry reconstruction and constitutive models adopted. All developed models were implemented in Abaqus/Standard 2019 (Dassault Systèmes Simulia Corp., Johnston, RI USA) and solved using a static analysis. A summary of the computational cost of all simulations, including the number of elements and wall-clock time, is provided in Table S2 of the Supplementary Materials.

### Mesoscale models

#### Geometry reconstruction and meshing process

**Fig. 2:**
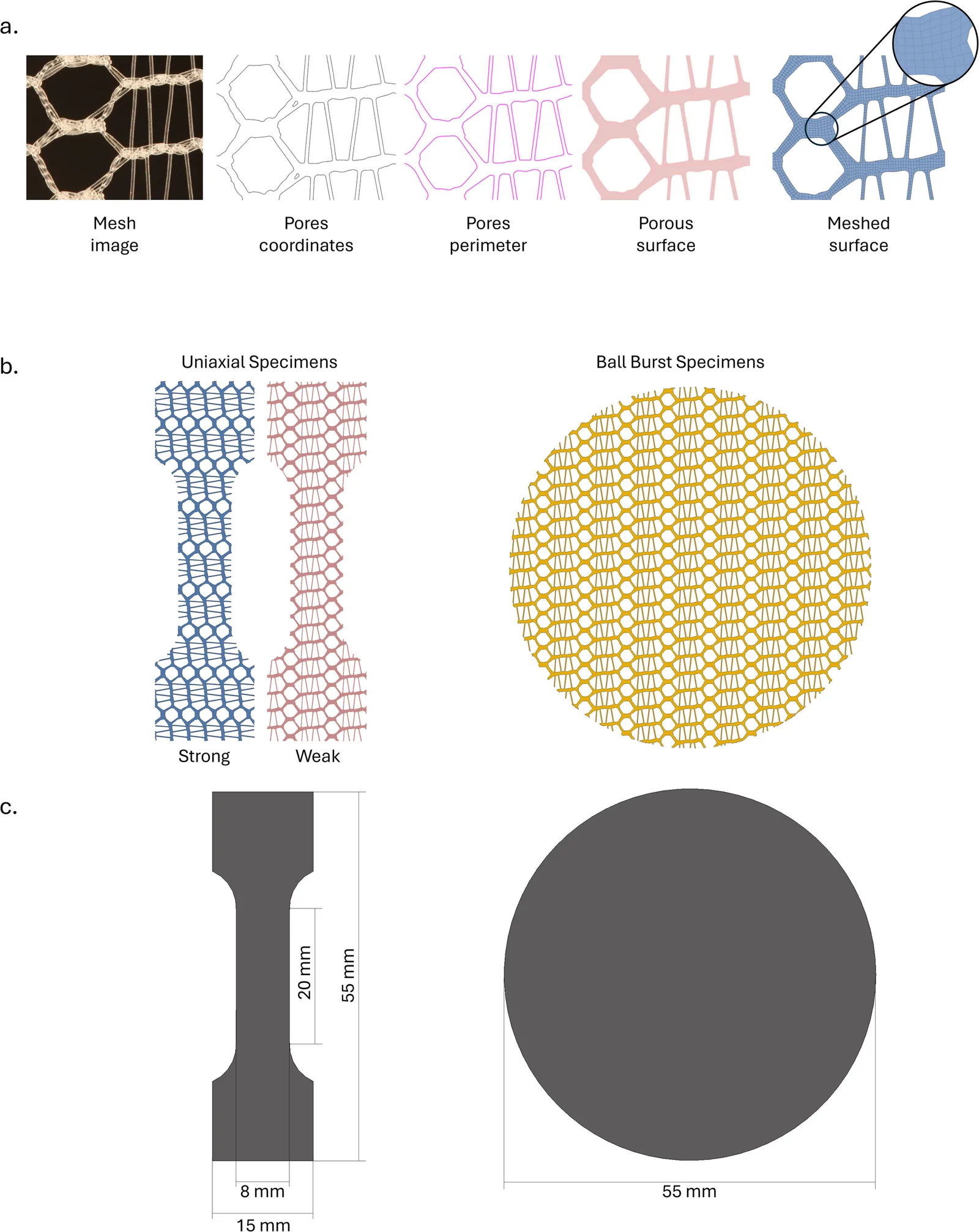
(**a**) Workflow from the 2D image to the meshed surface (Mesh L1). (**b**) Dogbone specimens, created for the two uniaxial tensile test directions, and the circular specimen for the BB test simulation in the mesoscale approach. (**c**) Dogbone specimen and the circular specimen for the BB test simulation in the macroscale approach

The workflow for the mesoscale porous architecture reconstruction is shown in Fig. 2a. The pores coordinates of a portion of each mesh were extracted from high-resolution binarized meshes images, acquired in a previous study, using the Matlab Image Processing Toolbox [38]. These coordinates were imported into the CAD software Rhinoceros 3D (Version 6.0, Robert McNeel & amp; Associates, Seattle, USA), where a unit cell was identified by isolating a representative area of the mesh structure. The perimeter of the pores within this unit cell was carefully delineated to reconstruct the porous surface geometry with high fidelity. In this approach, yarn intersections are represented as fully tied connections, without allowing for relative slip between yarns. This assumption was introduced to isolate the mechanical contribution of the pore architecture, neglecting additional effects associated with yarn-to-yarn sliding and local junction rearrangements, which would require a substantially more complex microscale reconstruction of individual filaments and their interactions. The meshing process was carried out in Hypermesh (Version 2019, CAE Altair Hyperworks, Troy, USA), using three-nodes S3 and four-nodes S4R shell elements as detailed in the mesh convergence analysis section of the Supplementary material.

The reconstructed and meshed unit cell was then repeated on the plane to create a patch, ensuring continuity by connecting adjacent nodes.

To replicate the in vitro tests (i.e., uniaxial tensile test and ball burst test), dogbone specimens aligned along the two principal knitting directions (weak and strong), and circular-shaped specimens were recreated with dimensions and orientations matching the experimental conditions Fig. 2b.

#### Constitutive material models

A hyperelastic material model was adopted to reproduce the nonlinear stress-strain behavior of these textile devices through the mesoscale FE models. In particular, a first-order Mooney-Rivlin isotropic model was selected among the hyperelastic constitutive models in Abaqus, after a previous evaluation detailed in the Supplementary materials [39, 40].

#### Thickness definition

**Fig. 3:**
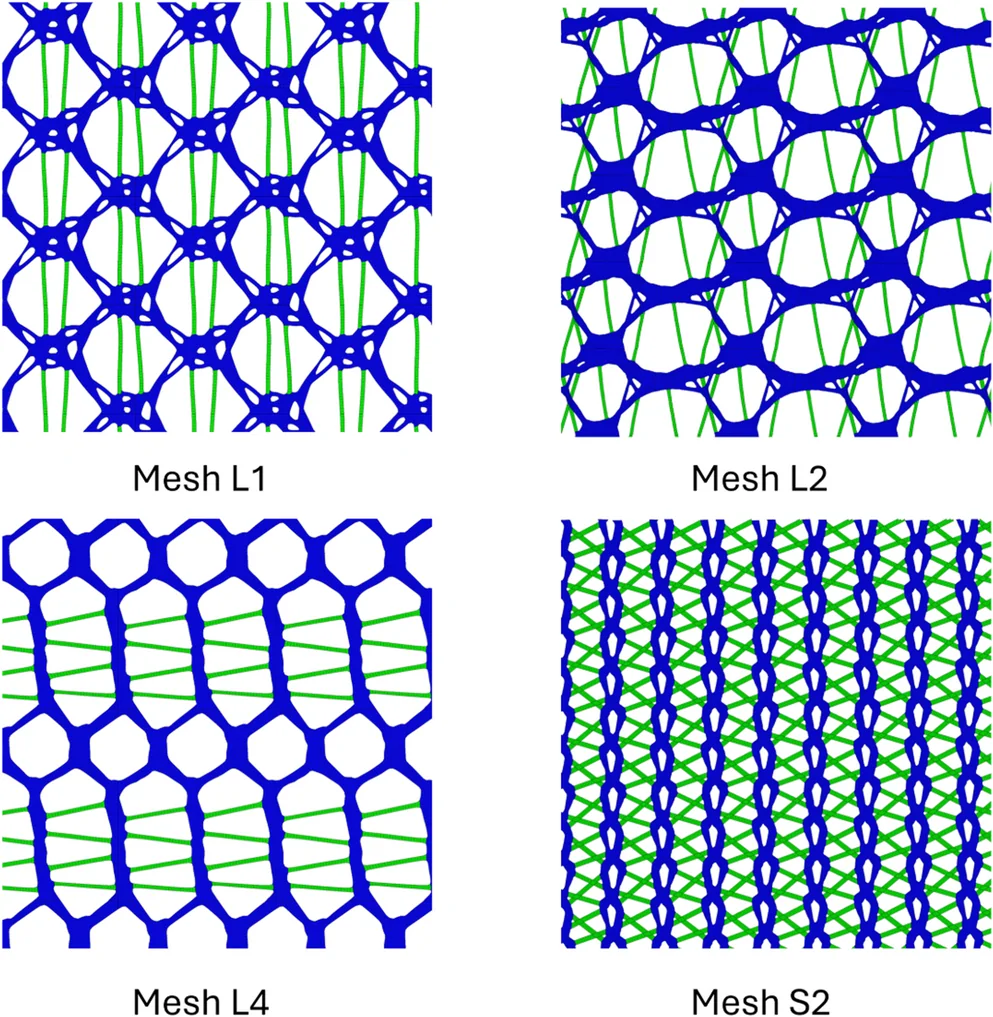
Representation of four meshes, with the single yarns set and the bundle set highlighted in different colors: green for the single yarns set and blue for the bundle set

In the meshes where it was possible to distinguish areas containing bundles of yarns (i.e., groups of interconnected yarns), and other areas consisting of single yarns that did not participate in fiber entanglements (L1, L2, L4, and S2, in Fig. 3), single yarns were isolated according to the following criteria:

1. Consecutive entanglements along a single yarn had to be separated by a distance greater than 1 mm.
2. A single yarn could not exhibit any intertwining along its length, although non-restrictive contacts with other fibers were allowed.

The specific thickness of the single yarns, experimentally measured using a thickness gauge, was assigned to the set of single yarns identified in this way. When these single yarns overlapped without forming nodes, they were allowed to slide and bend freely relative to one another. The overall mesh thickness, determined experimentally in a previous study using a thickness gauge, was then assigned to the set containing the yarn bundles [27]. The thickness assigned to the single yarns was 120 μm for the LW meshes and 180 μm for the SW mesh. The thicknesses used in the bundle part of mesoscale models are reported in Table 1.

### Macroscale models

#### Geometry reconstruction and meshing process

The geometries of the dogbone and circular specimens used for the uniaxial tensile tests and ball burst tests during the mechanical characterization campaign, respectively, were recreated in Rhinoceros 3D as a homogeneous surface (Fig. 2c). The surfaces were then meshed using both three-nodes S3 and four-nodes S4R shell elements in Hypermesh. Here, the overall thickness value was assigned to each mesh macroscale model.

#### Constitutive material model

An anisotropic hyperelastic constitutive material model was chosen for the macroscale models. Specifically, since the mechanical behavior of synthetic surgical meshes is strongly dependent on the knitted structure and, in particular, on the yarns direction, the Gasser-Ogden-Holzapfel (GOH) model was adopted [41]. The GOH model describes a fiber-reinforced hyperelastic material in which anisotropy is introduced through families of fibers characterized by a preferred orientation. In the present study, two fiber families symmetrically oriented with respect to the horizontal axis of the uniaxial specimens were considered (i.e., $\:\alpha\:$ and $\:-\alpha\:$) for all seven meshes. The model also includes a dispersion parameter $\:\kappa\:$, accounting for the degree of alignment of the fibers around the dominant direction. The parameter $\:\kappa\:$ can assume values in the range from 0 to 1/3. Lower dispersion values indicate that the fibers contribute more significantly along their primary direction, whereas higher dispersion results in a more isotropic behavior.

The fibers directions and their dispersion were phenomenologically calibrated together with the other material parameters within the calibration process. Other methods for the definition of the fibers directions were also analyzed and detailed in the Supplementary materials.

### Material calibration

#### FE analysis of uniaxial tests

**Fig. 4:**
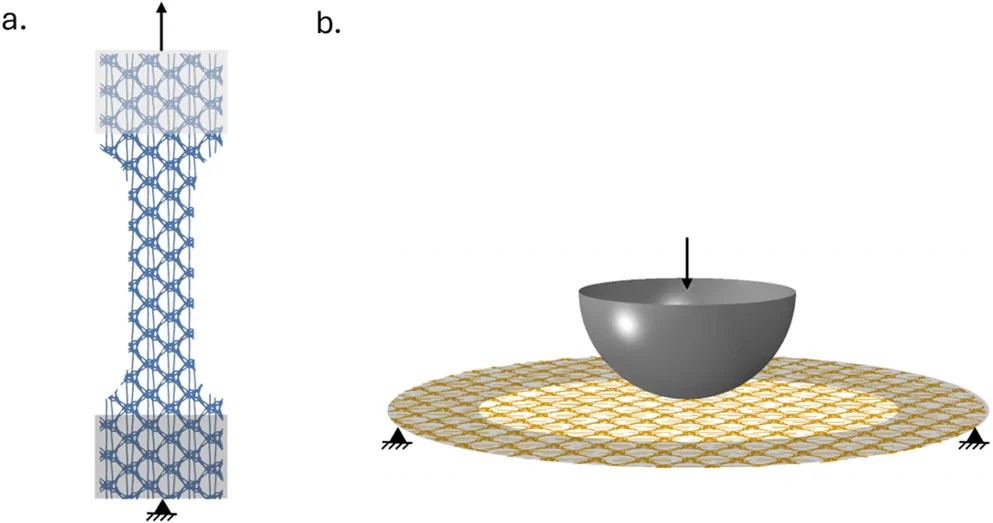
Boundary condition applied to replicate the in vitro testing setups. (**a**) Uniaxial tensile test: the darkest shaded area denotes the fixed nodes, while the lightest region indicates the nodes subjected to displacement. (**b**) Ball burst test: the darkest shaded area refers to the fixed nodes, while the vertical displacement was applied to the reference point located at the center of the hemisphere, kinematically coupled to its inner surface

Two reference points were defined and coupled to the lower and upper regions of the mesh using a kinematic coupling, replicating the pneumatic grip closure employed in the experimental tests. The lower region was then constrained with an encastre boundary condition, while the upper region was subjected to a vertical displacement, consistently with the displacement-controlled experimental protocol (20 mm/min) (Fig. 4a). The imposed displacement was set to achieve a 40% strain (i.e., 8 mm over a 20 mm gauge length).

#### Calibration of the material parameters

The calibration of the material was carried out in Hyperstudy (Version 2019, CAE Altair Hyperworks, Troy, USA) through a FE-based an inverse optimization process. Unlike analytical curve fitting approaches, where constitutive parameters are directly estimated by fitting an analytical function to the experimental response, the adopted methodology iteratively updates the material parameters based on the agreement between FE predictions and experimental results. At each iteration, the FE response associated with a specific parameter set was compared with the experimental outcomes and the material parameters were updated to minimize the objective function [42]. Herein, the objective function selected was the normalized root mean squared error, which was computed by summing the contributions of the vertical reaction force ($\:{F}_{y}$) from both knit directions at ten points along the curves:

1 $$f_{obj}\;=\;\sqrt{\frac{\sum_{i=1}^N\left[{\left(F_y^{exp}-\;F_y^{sim}\right)^2}_{is}\;+\;{\left(F_y^{exp}\;-\;F_y^{sim}\right)^2}_{iw}\right]}N}$$

$F_y^{exp}$ refers to the force registered by the load cell during the test, $F_y^{sim}$ refers to the force extracted at the upper region nodes at the end of the simulation. and refer to strong and weak direction, respectively.

In addition, the relative error of the force at 8 mm of displacement, computed as reported in Eq. 10, was used as a complementary metric for comparison.

2 $$e_{rel\_y}\;=\;\frac{F_y^{in\;silico}\;-\;F_y^{in\;vitro}}{F_y^{in\;vitro}}$$

where $F_y^{in\;silico}$ and $F_y^{in\;vitro}$ denote the simulated and experimental reaction forces, respectively.

Two different optimization algorithms were adopted in sequence: (1) a Global Response Surface Method (GRSM) and (2) an Adaptive Response Surface Method (ARSM) [43, 44].

GRSM is a surrogate-based global optimization approach that iteratively generates and evaluates multiple design points while adaptively updating the response surface approximation. New globally distributed designs are introduced at each iteration to maintain a balance between local search efficiency and global exploration capability. Since the optimization is performed on the approximated response surface rather than through direct gradient evaluation, GRSM enables an efficient exploration of the parameter space and can handle both single- and multi-objective optimization problems. Subsequently, ARSM was adopted as a local refinement strategy. ARSM constructs an initial response surface based on linear regression and progressively increases the approximation complexity as new evaluations are performed. The optimization proceeds iteratively until the convergence criterion is satisfied. The calibration followed an iterative cascade strategy, where the optimal parameter set obtained from each optimization cycle was used as the initial condition for the subsequent cycle until convergence of the objective function was achieved. A final refinement step using ARSM was performed to ensure local convergence.

#### Pore-level validation

**Fig. 5:**
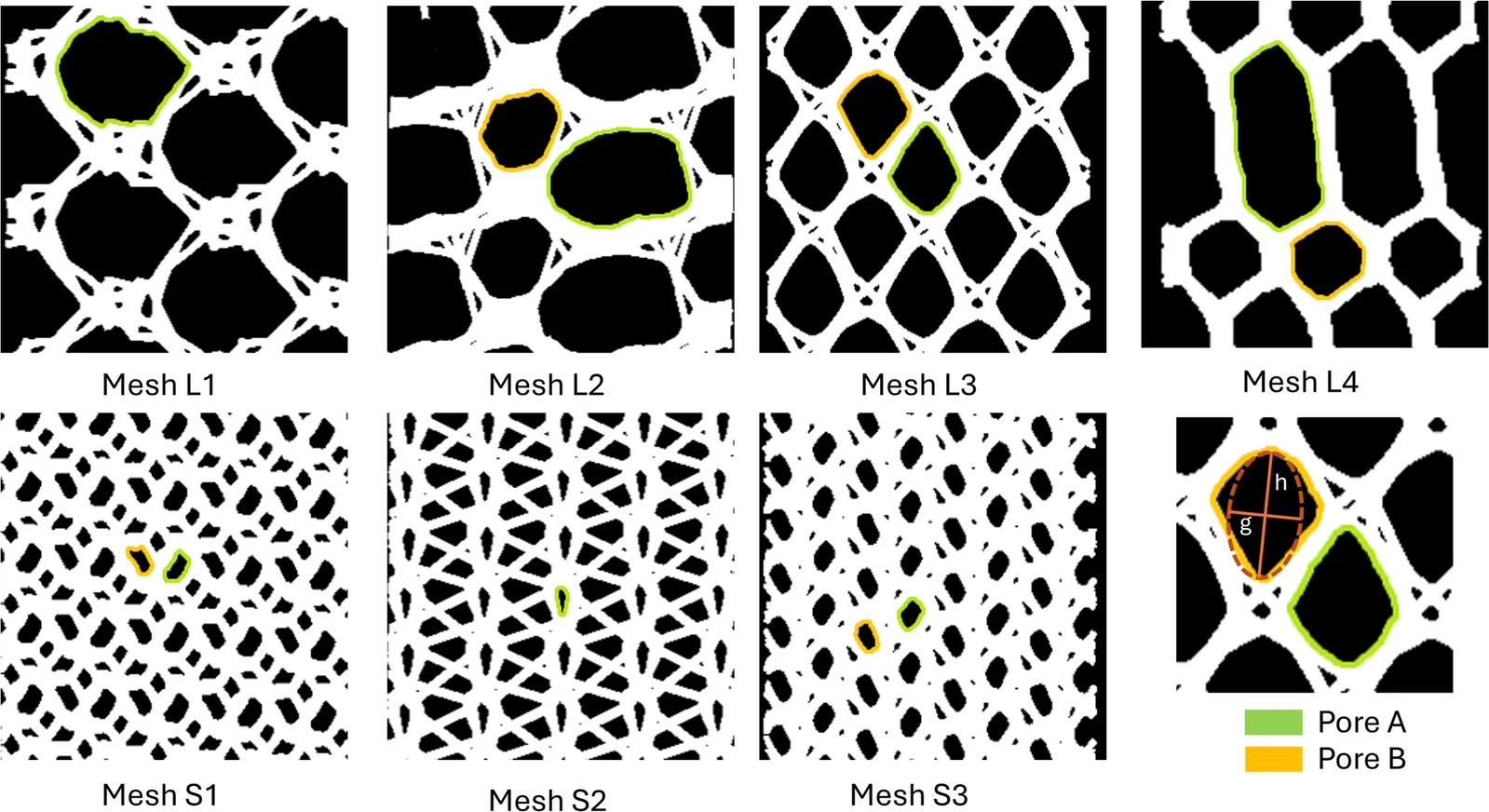
Representative binary images of the seven surgical meshes showing examples of the tracked pores. Pores A and pores B perimeters are highlighted in meshes L2, L3, L4, S1 and S3 while only pore A was identified in mesh L1 and S2. The image in the bottom right corner shows the geometric parameters used for the computation of the aspect ratio

A local analysis of the pores deformation during the uniaxial loading scenario for the mesoscale models was also carried out by means of image analysis. The images acquired during the in vitro uniaxial test used to compute the surgical mesh-level deformation of the specimens were binarized and processed using a semi-automated procedure in Matlab (version 9.10.0 (R2024a), Natick, Massachusetts: The MathWorks Inc.). For each mesh, pores with a similar configuration along the specimen were identified. Single yarns crossing the pores (Fig. 3) were not considered in this analysis, as their bending during the uniaxial test significantly hampers an accurate visualization and quantification of pore deformation. Two pore types were distinguished in five meshes (pore A and B in Fig. 5), while only pore A was identified in meshes L1 and S2. In detail, for each pore configuration, four pores along the specimen were analyzed; this number corresponds to the maximum number of comparable pores within the gauge section of the dog-bone specimens, ensuring consistency across all mesh types, including the more macroporous meshes.

The pore shape evolution was then tracked by analyzing successive images corresponding to applied displacements of 0, 2, 4, and 6 mm. Pores were matched across consecutive frames based on centroid proximity and morphological similarity, with the correspondence visually verified by the user to ensure accuracy. Morphological parameters such as pore area (* A)* and aspect ratio ($\:ar,\:ar=h/g)$ were extracted for each tracked pore, and the relative variation was computed as:

3 $$\triangle A\;=\;\frac{A_{final}\;-\;A_{initial}}{A_{initial}}$$

4 $$\triangle ar\;=\;\frac{ar_{final}\;-\;ar_{initial}}{ar_{initial}}$$

where $A_{final}$ and $ar_{final}$ correspond to the parameters computed from the images at 6 mm displacement whereas $A_{initial}$ and $ar_{initial}$ correspond to the parameters computed from the undeformed meshes.

The same procedure was applied to the frames extracted from the FE simulations to enable a direct comparison between numerical and experimental outcomes. For consistency, the same number of pores was analyzed, and the corresponding pore perimeters were extracted. The single yarns passing through the pores were not considered in this process because their bending during the uniaxial test makes the visualization of pores deformation very difficult.

### Validation with ball burst test

The consistency of the calibrated models was checked by replicating the testing conditions of ball burst tests previously performed on all the here analyzed meshes for both macroscale and mesoscale models [37]. The experimental tests were performed based on ASTM D6797-15 at a crosshead speed of 300 mm/min.

Figure 4b provides a schematic representation of the assembly and of the boundary conditions. The assembly was created by aligning the hemisphere with respect to the specimen center, with the dome of the hemisphere oriented towards the upper surface of the specimen. The steel sphere used during the test was modeled as a rigid hemisphere, defined as a *Discrete Rigid* body in Abaqus Standard. A surface-to-surface contact with hard normal contact and frictionless tangential behavior was defined between the rigid hemisphere and the mesh. To replicate the in vitro fastening through the cap of the ring, a circular ring of elements was fixed using an encastre boundary condition. The internal surface of the hemisphere was connected to a reference point defined in the center of the circular boundary through a kinematic coupling. This reference point was then displaced along the z-axis by 8 mm, indenting the mesh.

The force-displacement curves were extracted from the simulations and compared with the in vitro data to assess the behavior of the FE models under multidirectional loads and validate their accuracy using the area between in vitro and in silico results.

## Results

### Material calibration

#### Calibration of the material parameters

Table 2 reports the calibrated material parameters for both the macroscale and mesoscale approaches.

**Table 2:** Calibrated constitutive material coefficients obtained through the calibration procedure for each analyzed surgical mesh

Mesh ID	GOH parameters					Mooney-Rivlin parameters	
	alfa	C10	k1	k2	ki	C10	C01
L1	68.54	0.21	0.00	35.96	0.03	7.13	-3.52
L2	59.54	0.70	2.43	83.43	0.29	10.29	-3.45
L3	48.00	0.10	2.01	40.96	0.00	3.87	-0.61
L4	59.72	0.35	1.05	0.00	0.13	7.08	0.72
S1	44.09	0.31	0.00	0.00	0.14	2.17	-0.47
S2	66.72	0.33	5.69	56.32	0.28	4.55	1.13
S3	89.43	1.01	8.00	65.93	0.33	4.63	-1.75

**Fig. 6:**
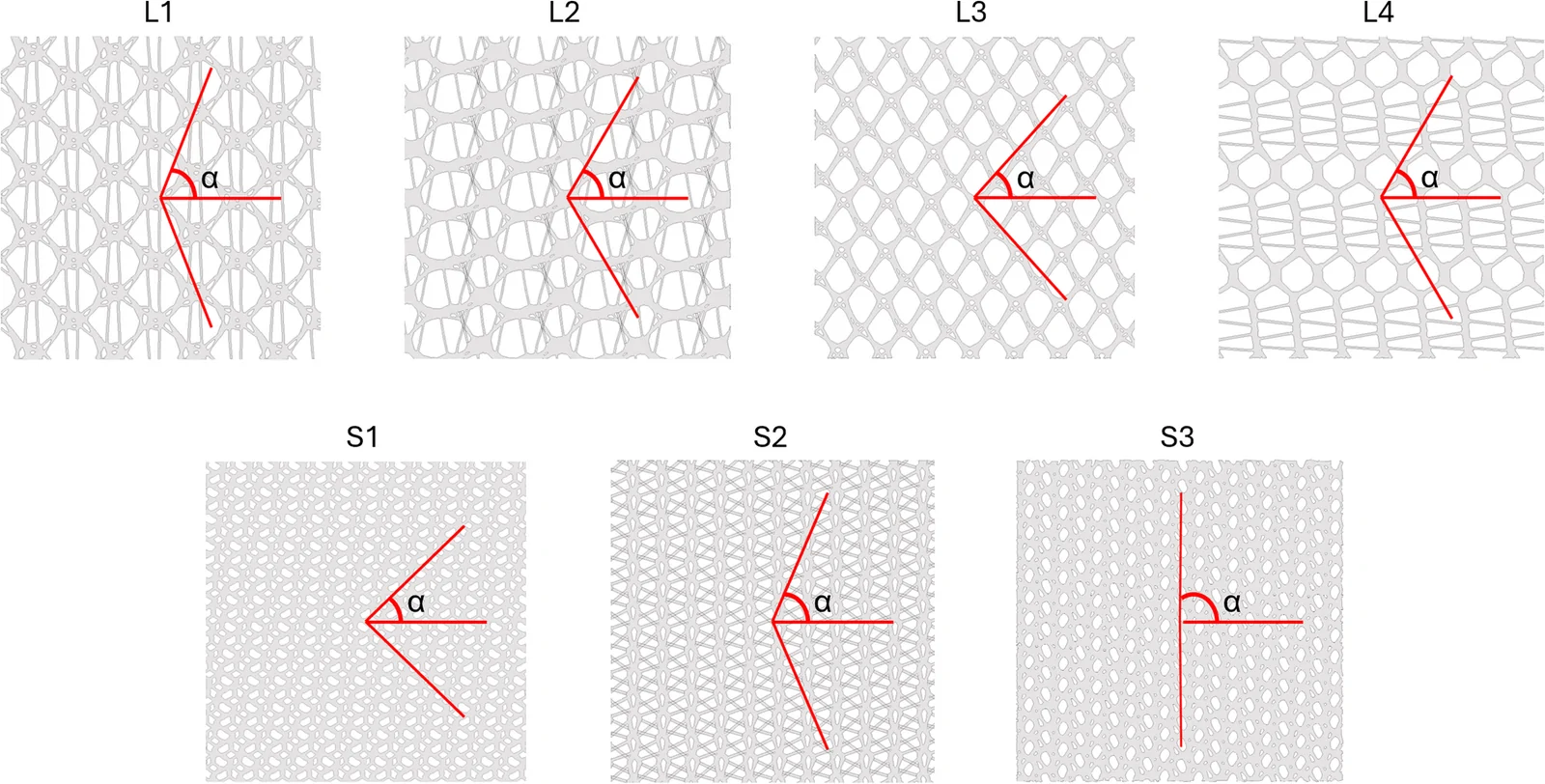
Reconstructed mesoscale meshes with the optimized fiber directions superimposed

The result of the calibration on the fiber orientations within the macroscale models is illustrated in Fig. 6, where the optimized directions are superimposed on the mesoscale geometries. Except for mesh L3, no clear correlation was observed between meshes geometry and the optimized directions. The optimized dispersions of the fibers suggest strong anisotropy only for two meshes (L1 and L3) and a quasi-isotropic behavior for three meshes (L2, S2, S3), with κ values approaching the isotropic limit (κ = 0.33).

**Fig. 7:**
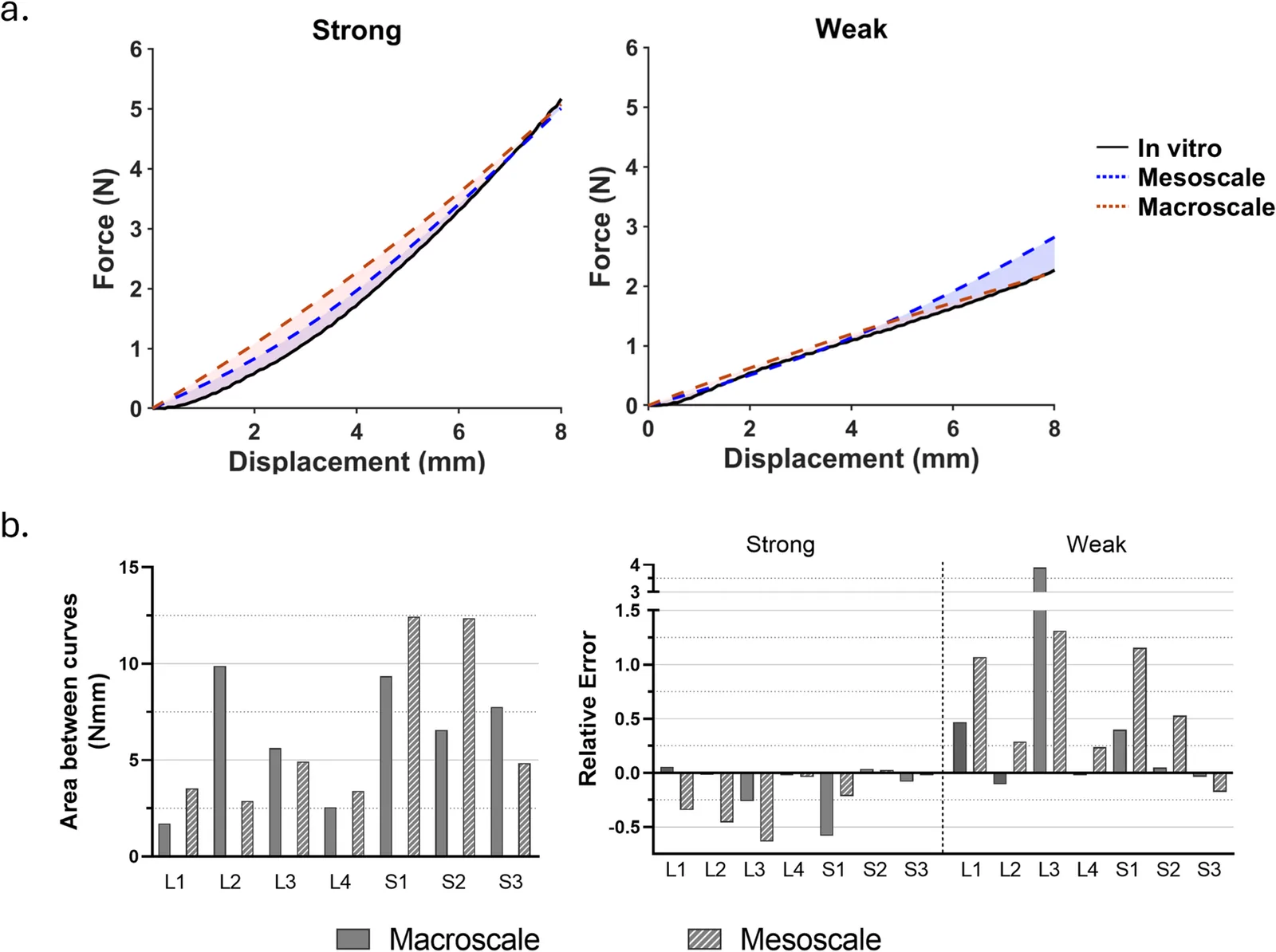
(**a**) Force–displacement curves of the L4 mesh, comparing experimental (in vitro) and numerical (in silico) responses. The shaded regions represent the area between the curves, used as a measure of their global dissimilarity: the light-blue regions refer to the comparison with the mesoscale model, while the red ones refer to the comparison with the macroscale model. (**b**) Comparison between the macroscopic and mesoscopic approaches in the material calibration. Left: total area between experimental and numerical curves, computed as the sum of the areas shown in panel a. Right: Relative error on the force at 8 mm displacement, computed for the same cases. Lower values indicate a better agreement between the numerical predictions and the experimental response

A representative force–displacement response of a mesoscale simulation following material calibration is shown in Fig. 7a, with the shaded regions highlighting the area between the in vitro and in silico force-displacement curves. Similar trends were obtained by computing the area between the curves through the two modelling strategies (Fig. 7b), with an overall advantage for the lightweight meshes, especially using the mesoscale approach. In the left panel, which reports the relative error of the force at 8 mm of displacement, the strong direction exhibited smaller error values than the weak direction. Together, these metrics provide a complementary assessment of both global and local agreement between simulations and experiments. The selected metrics are intended for comparative evaluation of modelling strategies rather than absolute validation against predefined error thresholds.

**Fig. 8:**
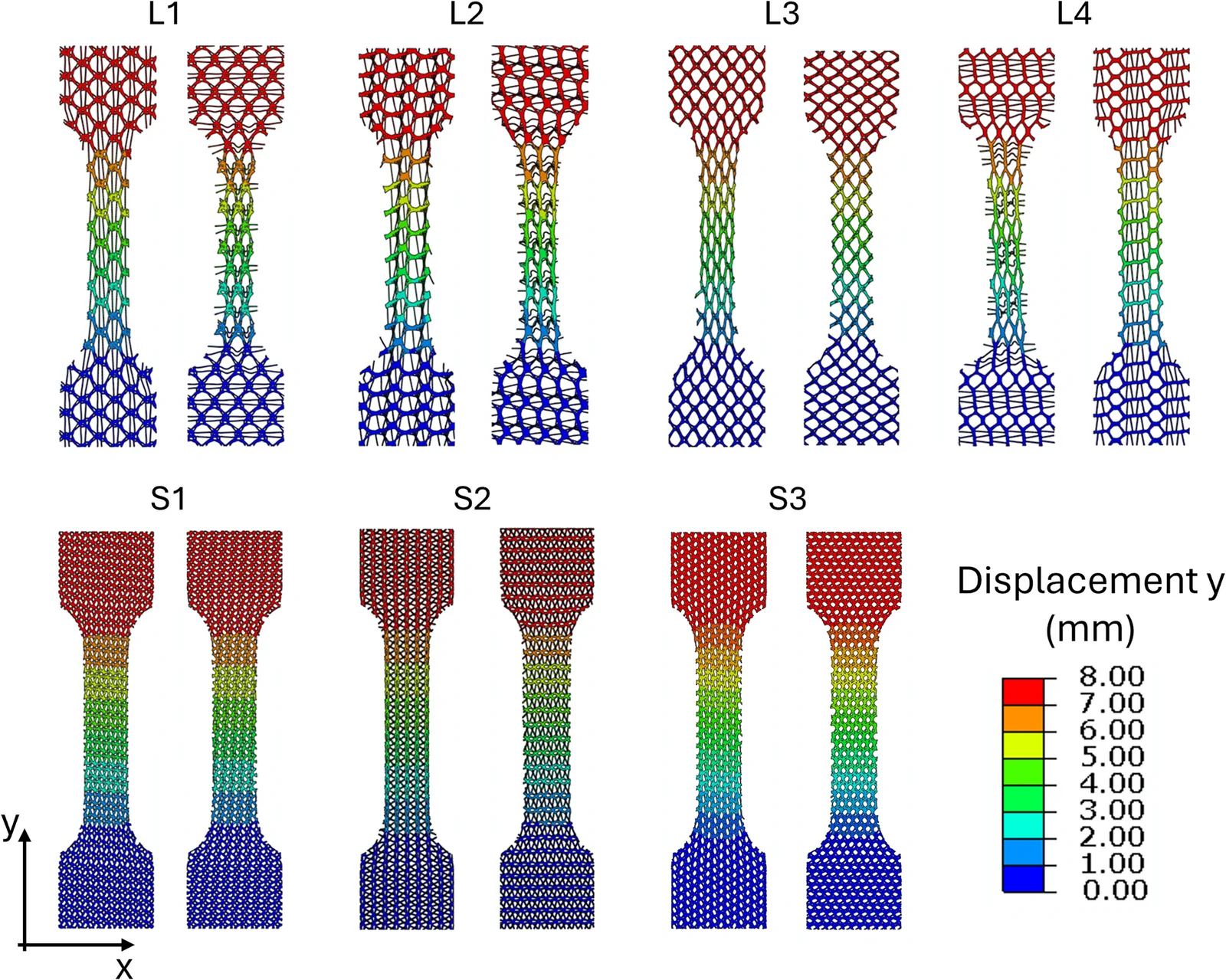
Deformed meshes subjected to 40% of strain. The contour plots depict the y-displacement

**Fig. 9:**
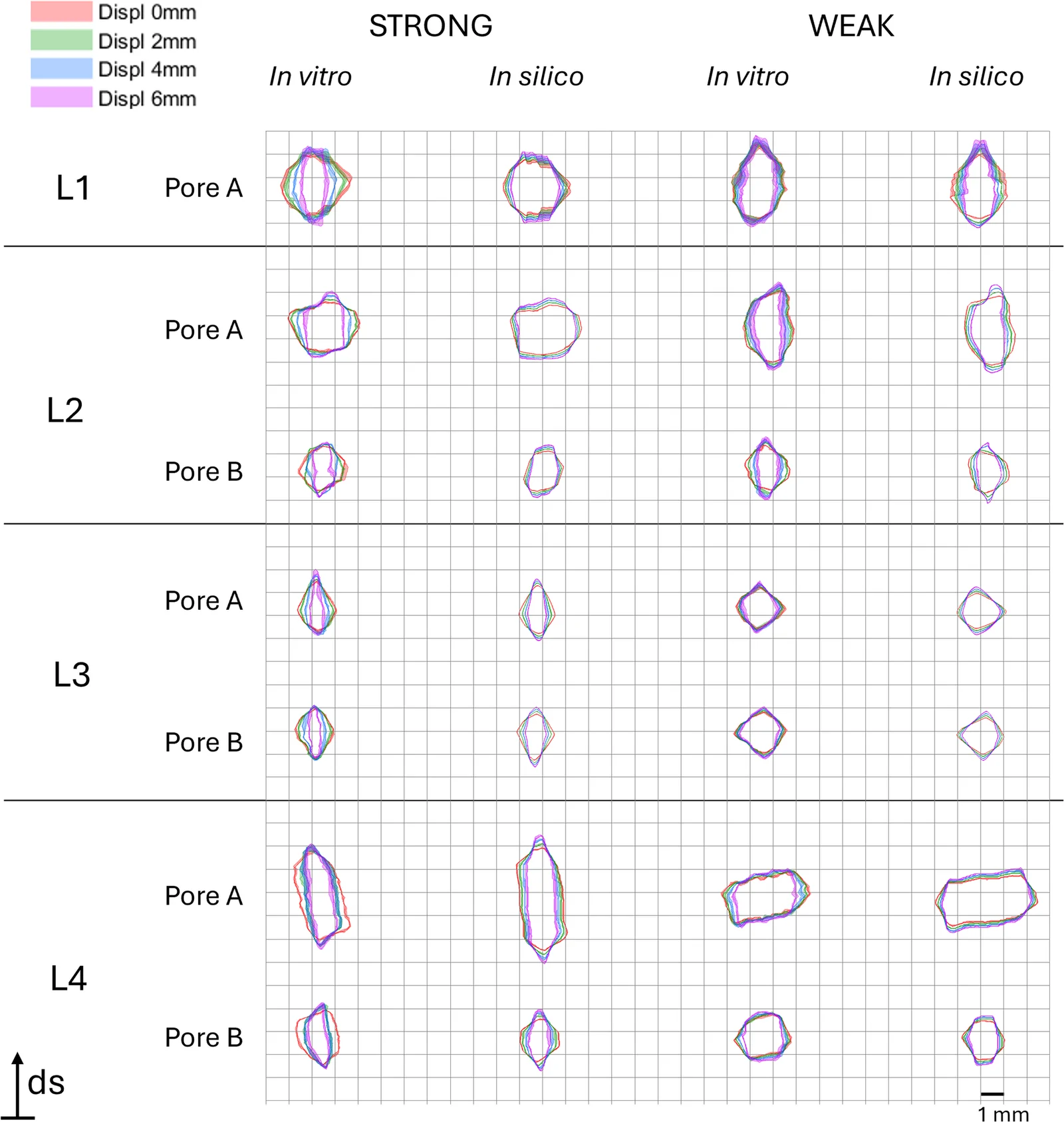
Pore shape deformation in the uniaxial test of LW meshes. Pore perimeters, represented as shaded areas, indicate the range of perimeter coordinates for each frame. Shaded regions corresponding to different displacement levels are superimposed and centered on the pore centroid to visualize the deformation evolution

**Fig. 10:**
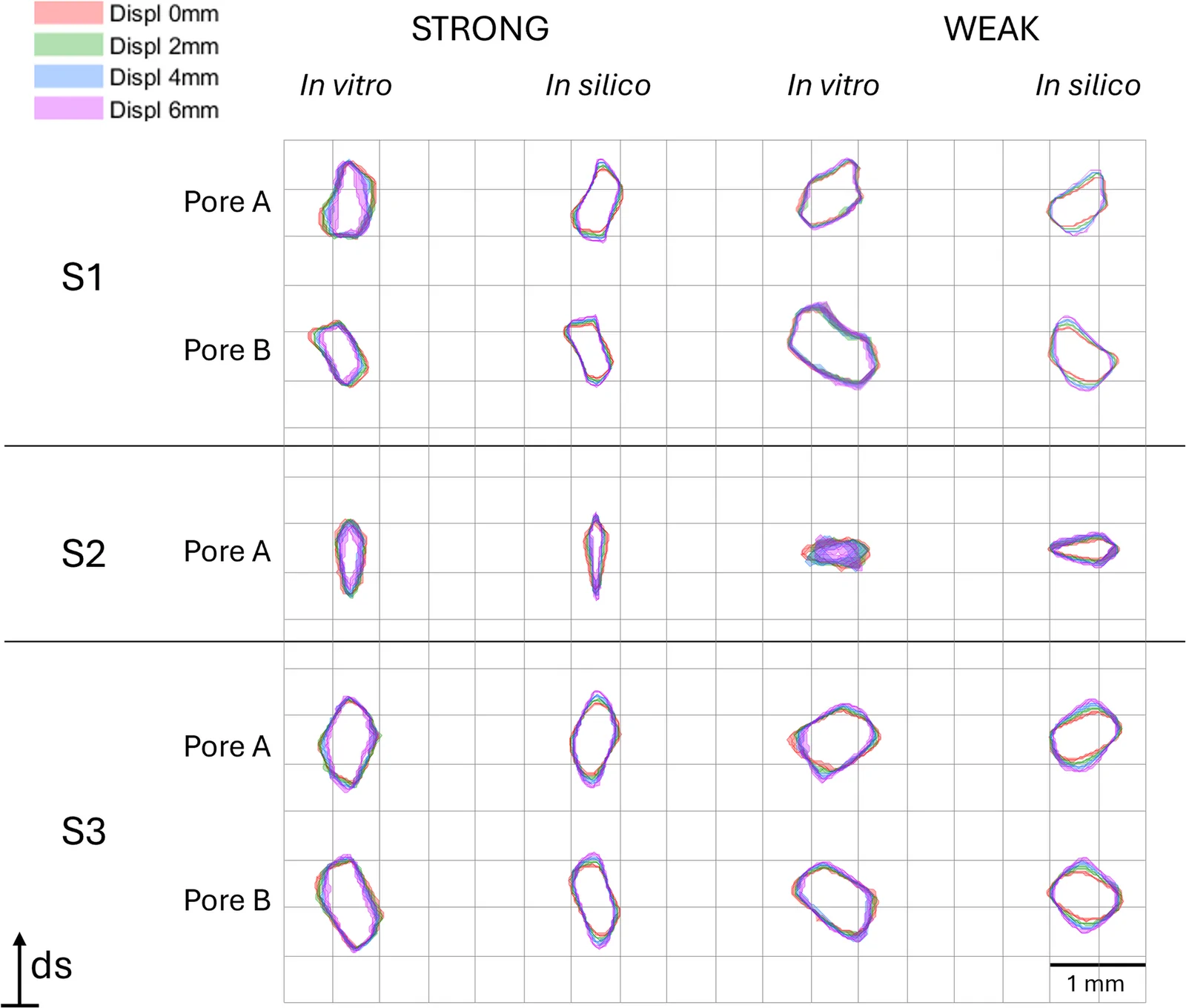
Pore shape deformation in the uniaxial test of SW meshes. Pore perimeters, represented as shaded areas, indicate the range of perimeter coordinates for each frame. Shaded regions corresponding to different displacement levels are superimposed and centered on the pore centroid to visualize the deformation evolution

Focusing on the mesoscale models, significant pore shape distortions were visible in the gauge length region at 8 mm of traction, particularly for the lightweight meshes (Fig. 8, upper row), due to pores collapse and a more pronounced lateral contraction during vertical displacement. A qualitative comparison between the in vitro and in silico deformed gauge regions is provided in the Supplementary Material, showing an overall good agreement in the predicted deformation patterns. This phenomenon is further discussed in the following paragraph and more clearly highlighted in Figs. 9 and 10.

#### Pore-level validation

**Fig. 11:**
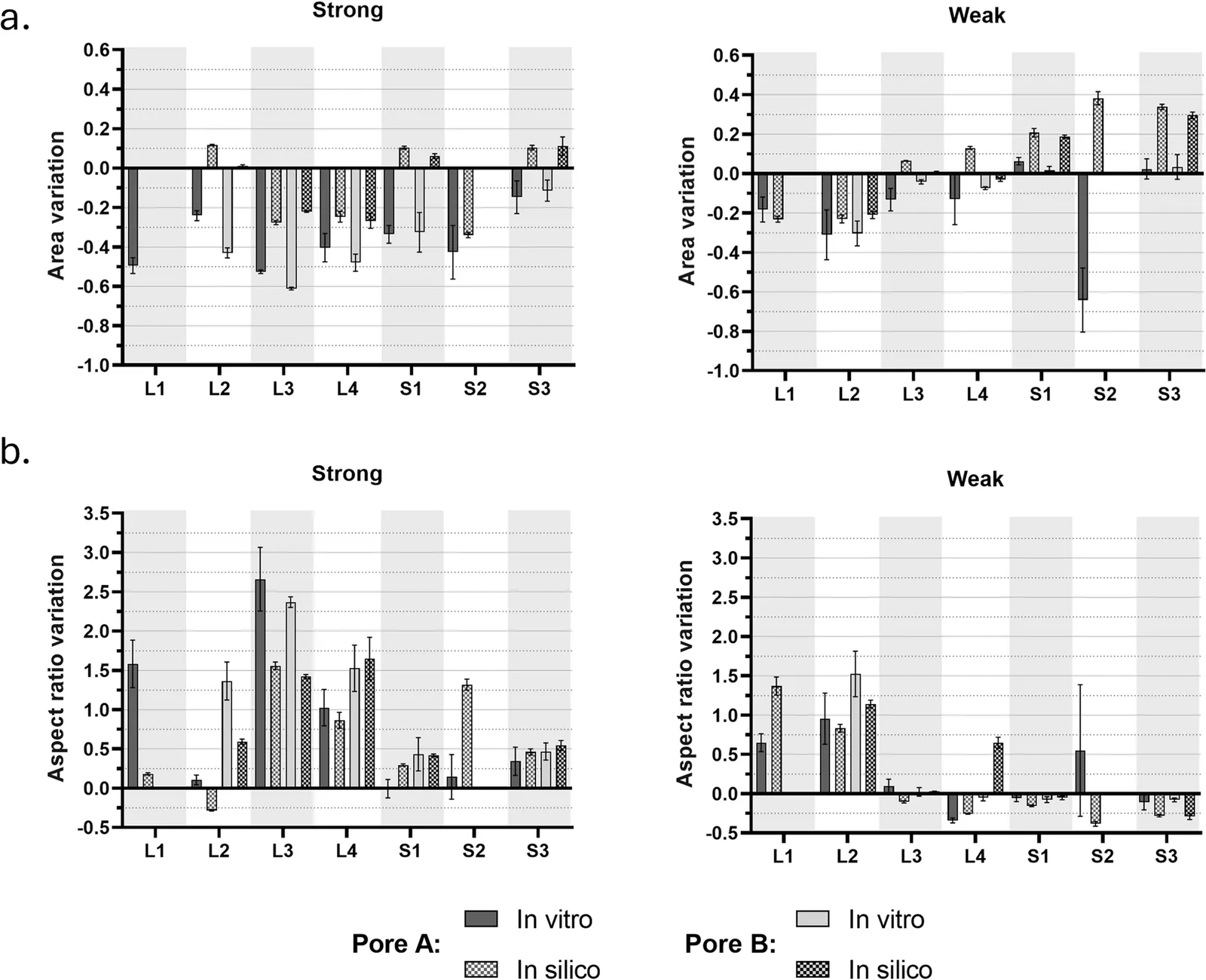
Parameters related to pore deformation under uniaxial loading. For each mesh, two different pores were analyzed (pore A and pore B), except for meshes L1 and S2, where only one pore type was considered. (a) Variation in pore area between undeformed and deformed shapes; (b) Variation in aspect ratio between undeformed and deformed shapes

Focusing on the pore-level analysis allowed by the mesoscale models, the negative values of the area variation parameter shown in Fig. 11 highlight a general collapsing trend for most meshes, especially in the strong direction, with reductions in area up to 60%. Pores elongate under loading, leading to increases in the aspect ratio values with peak values reaching 300% for mesh L3 in the strong direction. Collectively, the in silico models tended to underestimate pores deformation, both in terms of area and aspect ratio variation, across all meshes with the exception of mesh S3 and pores B of L4. Interestingly, a higher variability was observed in the in vitro analysis due to the variability in pores shape along the dogbone specimens.

The evolution of pores perimeters of the seven meshes was compared between the in vitro and in silico images for each displacement level (Figs. 9 and 10). The range of the perimeter coordinates identified for each frame has been represented as shaded areas centered on the pore centroid. The superimposition of these shaded areas allows for visualization of how pore deformation evolves between the undeformed and deformed configurations. The greater variability of the pores in vitro is clearly visible in the figure, particularly for mesh S2, where the pore in the weak direction completely collapses. Overall, a very good agreement can be appreciated between the experimentally measured deformed state and the model predictions, providing valuable insight into pore collapse under loading.

### Validation with ball burst test

**Fig. 12:**
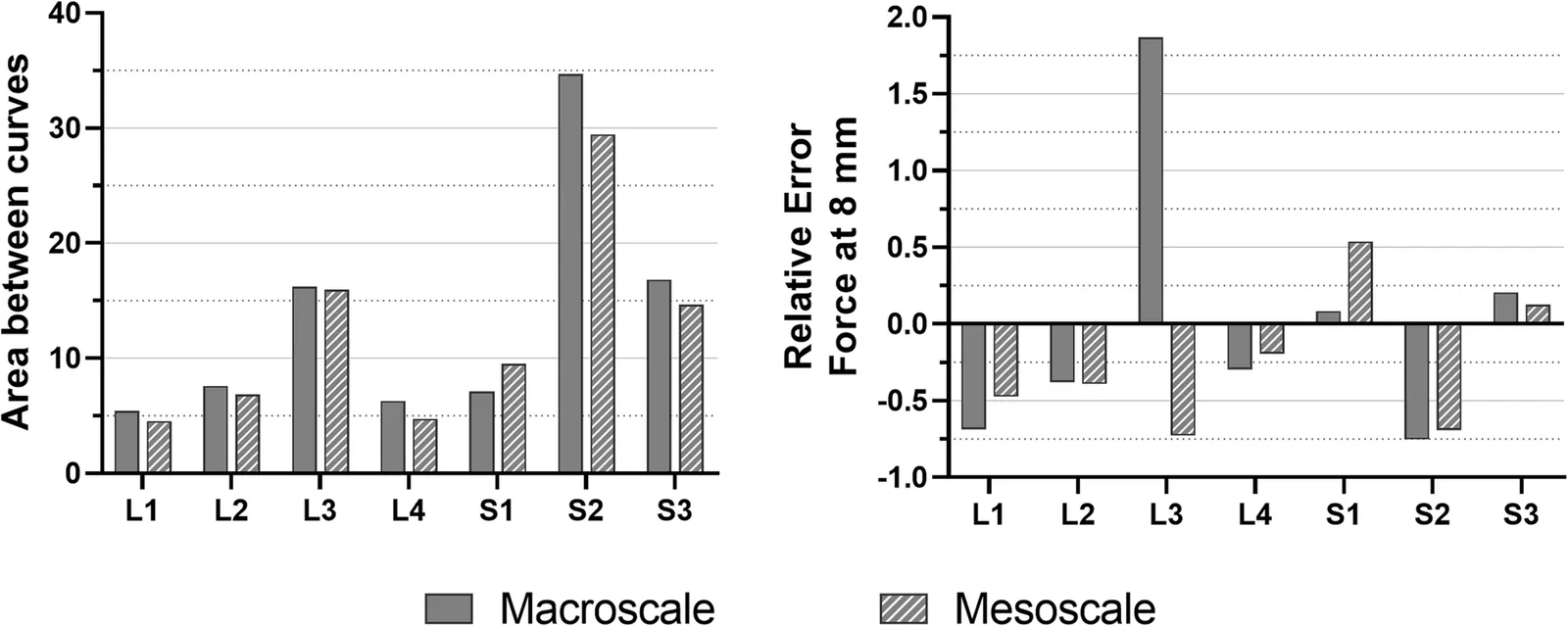
Left: Area between in vitro and in silico force-displacement curves for the optimized macroscale and mesoscale models in ball burst simulations after a sphere indentation of 8 mm. Right: Relative errors in the reaction force at 8 mm of indentation. The baseline at zero corresponds to the in vitro results. Bars above the baseline indicate an overestimation of the force, while bars below indicate an underestimation

The area between force-displacement curves computed for the ball burst simulations using both mesoscale and macroscale approaches is shown in Fig. 12 - Left. Similar values were observed for the same mesh simulated with the different approaches, with a slight overall advantage for the mesoscale approach. Additionally, the area values were in line with those obtained for the same mesh under uniaxial loading conditions.

A similar trend was observed for the relative error of the vertical force after 8 mm of hemisphere indentation (Fig. 12). Among all cases, meshes S3 and L4 exhibited the best agreement, while mesh L3 showed the largest discrepancies across both approaches. Specifically, for the mesoscale approach, the best-performing meshes were S3 and L4, whereas for the macroscale approach, meshes L2 and S3 yielded the most accurate results.

**Fig. 13:**
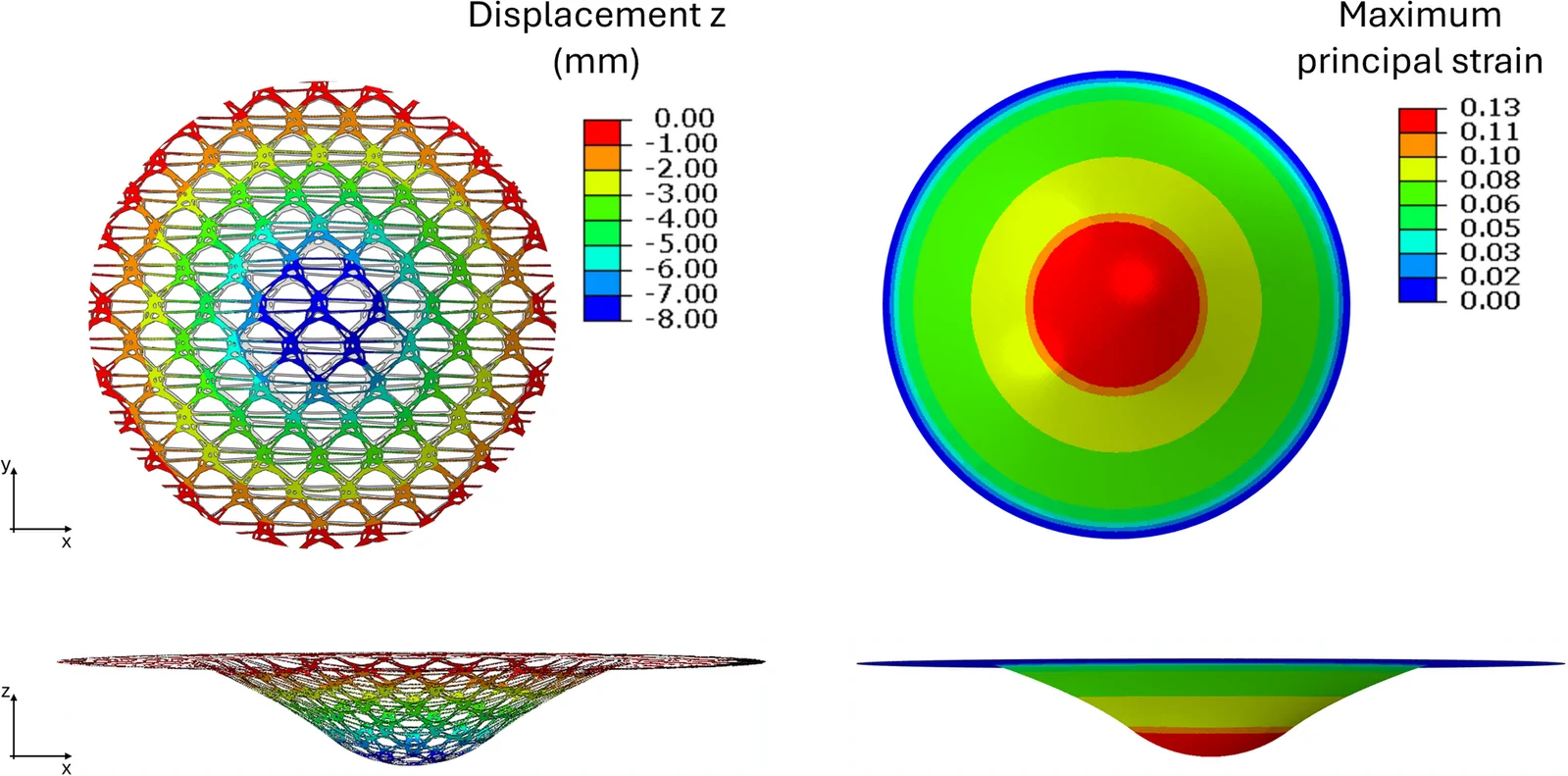
Deformed configurations of the L1 mesh in the xz-plane, with a magnified view of the central region shown in the xy-plane. Left: contour plot of the vertical displacement for the mesoscale model; the undeformed configuration is shown in grey in the magnified view. Right: contour plot of the maximum principal strain for the macroscale model

Figure 13 shows the contour plots of the deformed specimens modelled using both approaches after sphere indentation for the L1 mesh. The ability to capture pore shape deformation in mesoscale models (Fig. 13 – left) is clearly evident in the magnification that shows the undeformed specimen in grey with the superimposed contour plot of the enlarged mesh. Figure 13 – right shows the strain distribution obtained from the macroscale model, characterized by concentric regions with strain values reaching up to 13%.

## Discussion

In silico models of surgical meshes are crucial for predicting their behavior in vivo and elucidating the mechanisms behind mesh failure. However, full-device FE models typically do not incorporate the actual pore morphology, despite its key role in the interaction with abdominal tissues: rather, meshes are represented as homogeneous surfaces. To address this gap, we introduced a mesoscale modelling strategy that directly reconstructs the mesh architecture from high-resolution images and systematically compared it to a traditional macroscale formulation. By evaluating the models against experimental data, we assess how the level of morphological detail influences predictive accuracy. Hence, the implementation of FE models for polypropylene surgical meshes was conducted by replicating the most commonly used approach in literature, modeling surgical meshes as homogeneous surfaces with an anisotropic hyperelastic material formulation [20, 32, 45]. The lack of correspondence between the optimized fiber families of the GOH models and the actual knitted architecture, shown in Fig. 6, reduces the usefulness of these models in the design process. This highlights a fundamental weakness of macroscale formulations: by homogenizing the mesh and inferring fiber families through curve fitting, they may reproduce the overall response but fail to capture the true structural directions that govern the in vivo behavior of the device and that should be encountered in the designing phase. In this context, the calibrated fiber families in macroscale GOH models should be interpreted as effective anisotropy directions resulting from the inverse fitting procedure, rather than as direct representations of the underlying knitted architecture. This reflects the difference between the microstructural assumptions of the GOH constitutive framework and the architecture of textile-based surgical meshes. Originally developed for soft biological tissues such as arterial walls, where collagen fibers are embedded within an isotropic matrix with dispersed orientations, the GOH model is based on a fiber-in-matrix representation that is not directly representative of knitted polymeric structures. Nevertheless, the model remains effective in capturing the overall anisotropic mechanical response of the system. However, this discrepancy also limits the physical interpretability of the calibrated constitutive parameters, which cannot be directly associated with specific architectural features of the knitted mesh. As a result, while the homogenized model is suitable for reproducing the global mechanical behavior, it is less appropriate for guiding the structural design or optimization of textile-based surgical meshes, where explicit relationships between architecture and mechanical response are required.

On the other hand, morphology plays a major role in mesoscale models, where, despite using isotropic material definitions, the pores shape and orientation recreated through the porous surfaces were able to capture the anisotropic behavior. However, the mesoscale approach relies on a two-dimensional representation of an inherently three-dimensional knitted structure, thus neglecting out-of-plane features such as yarn interlacing and thickness effects.

As a result, both the macroscale and mesoscale approaches showed limitations in fully replicating the behavior of meshes with high anisotropic behavior. A clear example is provided by mesh L3 among the lightweight meshes, as well as mesh S1 and S2 among the standard weight meshes.

While fully resolved microscale models could, in principle, provide a more detailed representation of the knitted architecture, they are typically associated with high computational costs, complex contact definitions, and additional assumptions at the filament level. Moreover, such approaches are often not feasible for large-scale applications and do not necessarily translate into improved predictive capability of the mesh-level response [31, 46, 47].

Therefore, mesoscale models can be considered a reasonable compromise between physical representativeness and computational efficiency.

In this regard, the comparison between in vitro and in silico pore deformation revealed that the in silico models systematically predicted smaller deformations than those observed experimentally. This discrepancy highlights a key limitation of the proposed mesoscale framework: despite capturing the porous geometry of the meshes, the models do not explicitly represent the textile architecture and therefore neglect yarn-to-yarn sliding or local node loosening, mechanisms that can occur in the physical mesh under uniaxial loading and lead to more pronounced pore deformation. In the present mesoscale formulation, yarn intersections are modeled as fully tied in order to ensure a consistent representation of the knitted architecture at the structural (yarn-bundle) scale. This assumption avoids introducing additional degrees of freedom associated with filament-level interactions and maintains computational efficiency. In contrast, the real knitted mesh specimens may experience local fiber rearrangements, sliding, or rapid pore collapse once a critical geometry is reached, all of which increase the overall deformation. However, allowing such mechanisms would require an explicit microscale representation of individual filaments together with contact or cohesive interactions at yarn interfaces, significantly increasing model complexity and computational cost. It should be noticed that the visual superposition of pore perimeters at successive displacement steps, as employed here, represented a novel comparative approach: by centering the shaded ranges around each pore’s centroid and overlaying the frames, a direct, intuitive visualization of pore collapse and deformation evolution could be provided, reinforcing the quantitative metrics. While previous studies on surgical meshes have observed changes in pore size/shape or porosity under load (e.g., reduction of pore diameter under tensile load in prolapse meshes), we developed a detailed frame-by‐frame morphological tracking of individual pores in both experiment and simulation [6, 17, 35, 36, 48, 49]. Due to the test setup, it was not possible to acquire meshes images during the ball burst test, and therefore, this procedure could not be validated under an additional loading scenario.

Clinically, the ability of a surgical mesh to maintain its pore architecture under load is of high relevance: dramatic pore collapse can compromise tissue ingrowth, increase scar formation, affect load distribution, and ultimately contribute to complications such as hernia recurrence, mesh contraction, or fixation failure. Indeed, it has been shown that textile properties such as porosity and pore size under mechanical loading influence postoperative outcomes in hernia repair and pelvic mesh applications [7, 50, 51]. Therefore, our combined experimental–computational visualization offers a valuable tool for assessing mesh designs and may support improved design criteria or surgical placement strategies aimed at enhancing long-term clinical performance.

As for the ball burst test simulations, interestingly, the meshes with a lower error value after the calibration process showed the best performance. The main limitation of the macroscale models becomes evident when observing the contour plots during sphere indentation (Fig. 13). The strain patterns in the meshes do not reflect the directions of the fiber families and appear perfectly symmetrical, even though the low value of the optimized dispersion for the L1 mesh suggests anisotropic behavior. Furthermore, as noted above, since the optimized fiber direction is not linked to the actual morphology, it does not provide insight into the true strain distribution. In contrast, the pore dilatation captured by the mesoscale models offers specific insights into the local deformation behavior of the mesh under load. This pore expansion directly reveals how the structure redistributes strain, highlighting potential zones of stress concentration that contribute to the mesh’s overall mechanical response.

## Conclusion

This study proposes a new mesoscale approach based on the geometric replication of the porous architectures of lightweight and standard weight meshes. The approach was tested on seven meshes and compared to the conventional macroscale approach to evaluate the differences in terms of accuracy, effectiveness, and applicability. The macroscale approach is easy to implement but requires the implementation of an anisotropic hyperelastic material and completely neglects the knitted pattern. In contrast, the mesoscale approach, which incorporates morphological details, requires a longer pre-processing phase, particularly regarding the geometric reconstruction, but demonstrates a great ability to capture the anisotropic behavior of the meshes using isotropic materials.

Furthermore, it emerged that calibrated mesoscale and macroscale simulations achieved similar performances even in the ball burst test simulations, indicating that both approaches can be useful depending on the application context. In this context, the mesoscale models provide valuable insight into the pore-level deformation behavior under load, suggesting the possibility of integrating them into more complex models (e.g., a herniated abdominal model) or under multifaceted loading conditions.

These findings underscore the importance of incorporating morphological details into FE models to better understand the mechanical behavior of surgical meshes, providing a solid foundation for future research and practical evaluations before production. 

## Supplementary Information

Below is the link to the electronic supplementary material.

Supplementary Material 1 (DOCX 9.56 MB)
