# Supplementary material for: Comparative finite element modelling of surgical meshes at macro and mesoscale: assessing the role of morphology on mechanical behavior
Source: Med Biol Eng Comput. 2026 Aug 6;64(9):3471–88. doi: 10.1007/s11517-026-03645-6 (PMC13635049; doi:10.1007/s11517-026-03645-6)
Supplement: Supplementary file 1 [file 11517_2026_3645_MOESM1_ESM.docx]

# **Supplementary material**

for

**Comparative Finite Element Modelling of Surgical Meshes at Macro and Mesoscale: Assessing the Role of Morphology on Mechanical Behavior**

Vittoria Civilini^1,2^, Alessandra Aldieri^3^, Dario Carbonaro^1,4^, Vincenzo Giacalone^1,2^, Alberto L. Audenino^1,2^, Mara Terzini^1,2^

^1^ Department of Mechanical and Aerospace Engineering, Politecnico di Torino, 10129 Turin, Italy

^2^ Polito^BIO^Med Lab, Politecnico di Torino, 10129 Turin, Italy

^3^ Department of Industrial Engineering, Alma Mater Studiorum - University of Bologna, 40126 Bologna, Italy

^4^ Department of Mechanical, Energy and Management Engineering, Università della Calabria, 87030 Rende, Italy

## Mesh convergence analysis

In both the macroscale and mesoscale models, four-node (S4R) and three-node (S3) shell elements were employed.

Reduced integration was adopted to mitigate shear locking, while built-in hourglass control in Abaqus was used to prevent spurious zero-energy deformation modes. No numerical instabilities or non-physical deformation patterns were observed in the simulations. Geometric nonlinearities were included in the simulations (NLGEOM), enabling a finite deformation framework suitable for the large deformations observed experimentally.

To ensure the robustness of the numerical results, mesh convergence analyses were performed for both modeling approaches. In particular, six meshes with increasing element density were considered, and simulations were carried out using a linear elastic constitutive model to isolate discretization effects. At the macroscale, convergence was assessed by comparing the reaction force obtained with progressively refined meshes. At the mesoscale, both the global response and the local deformation of the pore architecture were evaluated. The global element size used in the different cases is reported in Table S 2.

Table S1: Number of elements in the convergence analyses for macroscale and mesoscale models

|  |  | **Number of elements** | |
| --- | --- | --- | --- |
|  |  | Macroscale | Mesoscale |
| **Global edge length (mm)** | 3 | 80 |  |
|  | 2 | 170 |  |
|  | 1 | 610 |  |
|  | 0.5 | 2498 |  |
|  | 0.3 | 6876 | 186 |
|  | 0.2 | 15399 | 360 |
|  | 0.15 |  | 557 |
|  | 0.1 |  | 1195 |
|  | 0.05 |  | 4612 |
|  | 0.025 |  | 18177 |

For the macroscale model, the convergence analysis was conducted by replicating the uniaxial tensile test. The bottom part of the dogbone specimen was fixed by applying an encastre boundary condition, while the upper part was kinematically coupled to a reference point, which was then vertically displaced by 10 mm.

Conversely, for the mesoscale model, only a unit cell of a single lightweight (LW) mesh was considered. This specific mesh, characterized by very large pores, was selected as the most critical for the meshing procedure. The boundary conditions applied were represented by an encastre constraint at the bottom nodes of the mesh and a 1 mm displacement imposed on the upper nodes.

Fig. S1a illustrates the relationship between element length, error, and computational cost, demonstrating the trade-off and the selected optimal element sizes.

Fig. S1: Mesh convergence analysis for the macroscale and mesoscale finite element models. a.: Percentage error on the vertical reaction force (RF, red line) and computational time (blue line) as a function of the element edge length. b.top: deformed configurations of the representative mesoscale unit cell obtained with progressively refined meshes. b.bottom: magnified views highlighting the local deformation of the pore architecture and individual yarns.

The convergence error was quantified as the relative difference between the RF obtained for a given mesh discretization and that of the finest mesh, assumed as reference:

$$error RF \left( \% \right)= \frac{{RF}_{i}-{RF}_{best}}{{RF}_{best}}*100$$

Eq. S1

To balance accuracy and computational efficiency, an element dimension of 0.5 mm was selected for the macroscale models, based on low error values and overall reduced computational time. For the mesoscale models, the computational costs appear manageable in the graphs due to modeling only a single unit cell. However, it is important to note that these costs will increase significantly when modeling entire specimens, resulting in much higher computational demands. Therefore, a relative error between the RF below 2% was deemed acceptable, leading to the use of element sizes with edge lengths of 0.1 mm (L1, L4, S3) and 0.05 mm (L2, L3, S1, S2). In addition to the global convergence assessment based on the reaction force, the influence of mesh refinement on the local deformation of the pore architecture was also evaluated (Fig. S1). As the mesh was progressively refined, only minor differences were observed in the deformation of individual yarns, whereas the predicted pore morphology and deformation remained essentially unchanged from the mesh with an element edge length of 0.1 mm onwards. Although some thinner yarns were represented by a single element through the thickness, the convergence analysis indicated that this discretisation did not significantly affect the predicted pore deformation, which was the main quantity of interest in the proposed mesoscale framework. Therefore, this mesh density was considered sufficient to ensure an accurate description of the local kinematics while maintaining a reasonable computational cost.

## Constitutive models comparison

### Macroscale models

Three different methodologies were developed to determine the direction of the fiber families: (1) a manual approach, (2) a histogram-based approach, and (3) an optimization-based approach. The first two methodologies were implemented, aiming to partially incorporate the geometry of the meshes into the macroscale model, while the optimization method reflects the most widely implemented approach for modeling surgical meshes.

Specifically, for the manual method and the histogram method, the images of the mesoscale unit cell was imported into Fiji (based on ImageJ, Schindelin et al., open-source, USA) . For the manual method, the number of fiber families was determined through visual inspection and the fiber directions were measured manually, assuming no dispersion (i.e., dispersion parameter $\kappa$ set to 0). Conversely, for the histogram-based method, the unit cell was first binarized and then skeletonized using the Skeleton plugin in Fiji. A directionality analysis was performed in order to extract the orientation of the fibers' skeleton lines using the Fourier component method. The resulting histogram, reporting the distribution of angles in degrees, was then exported and post-processed in Matlab. In the histogram graph, fiber families were identified as clusters of tall bars. Each cluster was subsequently inspected to determine the peak angle and the fibers dispersion for each family. The dispersion parameter for the model was set as the maximum value obtained across all fiber families of the same mesh. Lastly, for the optimization-based method, the fibers' directions included in the material model through the invariants $\bar{I}_{4\left( \alpha\right)}$ were optimized together with the other material parameters within the optimization phase. In this case, two fiber families were always considered (i.e., $\gamma$ and $-\gamma$ ).

### Mesoscale models

Two isotropic hyperelastic material models were adopted to reproduce the nonlinear stress-strain behavior of these textile devices through the mesoscale FE models. In particular, the Neo-Hookean and Mooney-Rivlin models were selected among the hyperelastic materials available in Abaqus. They differ in terms of the strain energy function and, consequently, the number of parameters that need to be defined in the input file. The two strain energy functions are detailed below:

$$W_{Neo-Hookean}= C_{10}\left( \bar{I_{1}}-3 \right)+\frac{1}{D}{(J-1)}^{2}$$

Eq. S2

$$W_{Mooney-Rivlin}= C_{10}\left( \bar{I_{1}}-3 \right)+C_{01}\left( \bar{I_{2}}-3 \right)+\frac{1}{D}{(J-1)}^{2}$$

Eq. S3

Where:

$\bar{I_{1}}, \bar{I_{2}}$: First and second invariant of the Cauchy-Green strain tensor

$J$: Determinant of the deformation gradient (volumetric deformation)

$C_{10}, C_{01}$: Materials parameters

$D$: Parameter related to bulk modulus (incompressibility)

### Material models comparison

The comparison among the constitutive material models for mesoscale and macroscale models was performed by evaluating the area between *in vitro* and *in silico* force-displacement curves.

Fig. S2 depicts the area between *in vitro* and *in silico* force-displacement curves. For mesoscale models, lower area values were attained by Mooney-Rivlin models in five meshes and slightly higher in two. Comparing the methods in determining the fibers orientation, the worst results were obtained using the histograms method with higher differences for meshes ID 4 and 8. Generally, better agreement was achieved using the optimization method, with the exception of mesh ID 8, in which the lowest area value was obtained by the manual method.

Fig. S2: Area between in vitro and in silico force-displacement curves at 40% of strain for the optimized macroscale and mesoscale models

To identify inhomogeneity in simulating the two principal directions (i.e., strong and weak directions), the relative error of the vertical reaction force ($e_{rel\_y})$ was computed as:

$$e_{rel\_y}= \frac{F_{y}^{in silico}-F_{y}^{in vitro}}{F_{y}^{in vitro}}$$

Eq. 3

where $F_{y}^{in silico}$ and $F_{y}^{in vitro}$denote the simulated and experimental reaction forces, respectively.

Overall, no substantial differences were observed when simulating the strong and weak directions. Additionally, for several meshes, comparable results were obtained when considering the same scale and constitutive model. At mesoscale length, better results were gained by the Mooney-Rivlin formulation, while for macroscale models, similar values were obtained by manual and optimization methods. Comparable results were achieved at different length scale simulations, with the exception of the histograms method, which achieved the worst overall results. Comparing the meshes considering all formulations and length scales, meshes ID 6 and 10 had the best performance, while ID 4 had the worst, especially in the weak direction.

Fig. S3: Relative errors in the reaction force at 40% of strain. The baseline at zero corresponds to the in vitro results. Bars above the baseline indicate an overestimation of the force, while bars below indicate an underestimation

Comparison of in vitro and in silico gauge region deformation

Fig. S4 reports a qualitative comparison between the experimental Digital Image Correlation (DIC) images and the corresponding finite element predictions at 8 mm of imposed displacement. An overall good agreement is observed in the deformation pattern of the gauge region, particularly for meshes L2, L3, L4 and S3. For the remaining meshes, the numerical models generally predict a slightly lower deformation than that observed experimentally.

It should be noted that the DIC images were acquired for displacement tracking rather than for morphological characterization of the mesh architecture. Consequently, the visibility of individual yarns and pores is affected by the image contrast, which is not always optimal for a detailed comparison of the pore geometry. For this reason, a more accurate assessment of pore deformation is provided in Figs. 10 and 11 of the main manuscript.

Fig. S4: Qualitative comparison between the in vitro Digital Image Correlation (DIC) images and the corresponding in silico deformed configurations in the gauge region at 8 mm of imposed displacement for all the analyzed surgical meshes.

Computational Cost Analysis

All simulations were performed on a workstation equipped with an Intel Core i9-14900 processor (24 cores, 32 threads) and 64 GB DDR5 RAM. Table S2 reports the number of nodes and the computational cost for the uniaxial models. Computational cost is reported in terms of wall-clock time.

Table S2: Computational details for all simulations. The table reports the number of finite elements and the wall-clock time required for each simulation for both modelling approaches.

|  | **Macroscale** | | | **Mesoscale** | | | |
| --- | --- | --- | --- | --- | --- | --- | --- |
| **Mesh ID** | **Nodes number** | **Wallclock time (s)** | | **Nodes number** | | **Wallclock time (s)** | |
|  |  | **Strong** | **Weak** | **Strong** | **Weak** | **Strong** | **Weak** |
| L1 | 2633 | 83 | 77 | 30453 | 30385 | 393 | 552 |
| L2 |  | 95 | 100 | 122374 | 124892 | 447 | 1531 |
| L3 |  | 46 | 47 | 135503 | 135213 | 1390 | 1777 |
| L4 |  | 80 | 83 | 28972 | 79955 | 447 | 261 |
| S1 |  | 45 | 47 | 158310 | 157744 | 1548 | 1423 |
| S2 |  | 60 | 62 | 170053 | 167464 | 1601 | 1866 |
| S3 |  | 78 | 56 | 55561 | 55594 | 347 | 336 |
